# Perfil Clínico e Evolução de Pacientes com Infecção Relacionada a Dispositivos Cardíacos Eletrônicos Implantáveis

**DOI:** 10.36660/abc.20190546

**Published:** 2021-06-08

**Authors:** Alessandra de Souza Maciel, Rose Mary Ferreira Lisboa da Silva

**Affiliations:** 1 Universidade Federal de Minas Gerais Hospital das Clínicas Belo HorizonteMG Brasil Universidade Federal de Minas Gerais - Hospital das Clínicas, Belo Horizonte, MG - Brasil; 2 Universidade Federal de Minas Gerais Departamento de Clínica Médica Belo HorizonteMG Brasil Universidade Federal de Minas Gerais - Departamento de Clínica Médica, Belo Horizonte, MG - Brasil

**Keywords:** Marca-Passo Artificial, Procedimentos Cirúrgicos Cardiovasculares, Bactéria, Endocardite, Evolução Clínica, Infecção

## Abstract

**Fundamento:**

Houve aumento expressivo na incidência de infecções relacionadas a dispositivos cardíacos eletrônicos implantáveis (DCEI) nos últimos anos, com impacto na mortalidade.

**Objetivos:**

Verificar a proporção de pacientes com infecção de DCEI e analisar seu perfil clínico, as variáveis relacionadas com a infecção e sua evolução.

**Método:**

Estudo retrospectivo, observacional e longitudinal com 123 pacientes com infecção de DCEI entre 6.406 procedimentos. Foram usados os testes paramétricos, e o nível de significância adotado na análise estatística foi de 5%.

**Resultados:**

A idade média dos pacientes foi de 60,1 anos, e 71 eram homens. A média de internação foi de 35,3 dias, e houve remoção total do sistema em 105 pacientes. Identificaram-se endocardite infecciosa (EI) e sepse em 71 e 23 pacientes, respectivamente. A mortalidade intra-hospitalar foi 19,5%. Houve associação entre EI e extrusão do gerador (17,0% vs. 19,5% nos grupos com e sem EI, respectivamente, p = 0,04; associação inversa) e sepse (15,4% vs. 3,2%, p = 0,01). Houve associação entre morte intra-hospitalar e EI (83,3% vs. 52,0% com e sem morte, respectivamente, p = 0,005) e sepse (62,5% vs. 8,1%, p < 0,0001). Foi dada alta hospitalar a 99 pacientes. Durante a média de seguimento clínico de 43,8 meses, a taxa de mortalidade foi de 43%, e 65,2% dos pacientes com sepse faleceram (p < 0,0001). A curva de sobrevida de Kaplan-Meier não indicou associação significante com sexo, agente etiológico, fração de ejeção, EI e modalidade de tratamento. A taxa de mortalidade foi de 32,8% entre os pacientes submetidos a reimplante de eletrodos por via endocárdica e 52,2% entre aqueles por via epicárdica (p = 0,04). Não houve influência da etiologia chagásica, a qual correspondeu a 44,7% das cardiopatias de base, quanto às variáveis clínicas e laboratoriais ou à evolução.

**Conclusões:**

A taxa de infecção foi de 1,9%, com predomínio em homens. Houve associação entre mortalidade intra-hospitalar e EI e sepse. Após a alta hospitalar, a taxa de mortalidade anual foi de 11,8%, com influência de sepse durante a internação e o implante epicárdico. (Arq Bras Cardiol. 2021; [online].ahead print, PP.0-0)

## Introdução

O uso de dispositivos cardíacos eletrônicos implantáveis (DCEI) cresceu de forma exponencial nos últimos 10 anos devido ao avanço da tecnologia, à ampliação das indicações e à maior expectativa de vida. Por outro lado, durante esse período, houve um aumento importante e desproporcional (de 210%) na incidência de infecções relacionadas aos DCEI, alcançando a incidência de até 19,9%.^1-4^ Essas infecções estão relacionadas ao tipo de dispositivo e ao número de suas manipulações.^5^ Após a realização de substituição do dispositivo, o risco de infecção é de cerca de 5%, com um aumento de 2 a 4 vezes em comparação com o risco de um implante primário.^5,6^ Outros fatores também estão associados com o aumento da infecção, tais como sexo, idade, comorbidades e falta de profilaxia.^7,8^

A infecção relacionado com DCEI apresenta morbidade significativa e mortalidade intra-hospitalar que varia de 6 a 14%, com mortalidade total de aproximadamente 20% em um ano, incluindo o período após a alta hospitalar.^1,6,9^ Além disso, há variáveis associadas a desfechos desfavoráveis e preditores de mortalidade, como idade do paciente, uso de marca-passo (MP) temporário, trocas de dispositivos, agente estafilococo como etiologia, presença de prótese valvar cardíaca, tempo de remoção do dispositivo, insuficiência renal, necessidade de transfusão de sangue e presença de endocardite.^1,10-13^ O risco de morte por infecção do DCEI depende do tipo de dispositivo e persiste ao longo do tempo. A taxa de mortalidade de até 20% permanece durante 3 anos para MP de câmera única ou dupla e 2 anos para cardioversor-desfibrilador implantável (CDI).^14^

Em nosso meio, há escassa informação sobre esse tema, além do fato de as características dos pacientes e as etiologias para o implante de DCEI serem distintas dos países desenvolvidos. Portanto, o conhecimento do perfil desses pacientes e sua evolução são etapas iniciais importantes para a execução das diretrizes estabelecidas na literatura.^15^ À vista disso, os objetivos deste estudo foram verificar a proporção de pacientes com infecção relacionada a DCEI e analisar seu perfil clínico e laboratorial, as variáveis relacionadas com a infecção e sua evolução.

## Métodos

Trata-se de um estudo de coorte, observacional, longitudinal e retrospectivo. A população foi constituída por 123 pacientes com infecção relacionada a DCEI, de ambos os sexos e de todas as idades, no período de 2001 a 2017, considerando 6.406 procedimentos de implante de DCEI. Foram excluídos pacientes com infecção relacionada a MP temporário, e também foram excluídos pacientes com infecção do DCEI, porém submetidos ao implante do dispositivo em outros hospitais.Tanto o projeto de pesquisa quanto o termo de consentimento livre e esclarecido foram aprovados pelo Comitê de Ética em Pesquisa da instituição, em conformidade com o descrito na resolução 466/2012. Foram analisadas variáveis clínicas e laboratoriais e dados de tratamento farmacológico e não farmacológico. Para o diagnóstico de infecção relacionada a DCEI, foram considerados exames clínicos associados a hemograma, proteína C-reativa, hemoculturas e ecocardiograma. Para diagnóstico de endocardite infecciosa, foram utilizados os critérios de Duke modificados.^16^

A profilaxia e o tratamento das infecções relacionadas a DCEI realizados na instituição na qual foi desenvolvido o estudo incluíram técnicas assépticas com banho de clorexidene dergemante na noite anterior e na manhã da realização do procedimento, remoção de pelo, degermação cirúrgica, antissepsia da pele com clorexidine degermante durante 2 minutos e, após remover o excesso, aplicação de clorexidine alcoólica. Segundo o mesmo protocolo, a profilaxia antibiótica foi realizada 1 hora antes do procedimento com dose única de cefazolina 2 g.

O quadro de sepse foi definido como disfunção orgânica potencialmente fatal causada por uma resposta imune desregulada a uma infecção.^17^ Foram considerados mortalidade intra-hospitalar todos os óbitos ocorridos em razão da infecção no período de internação. Após a alta hospitalar, os pacientes sobreviventes foram acompanhados durante um período mínimo de 6 meses. Foram considerados morte após a alta hospitalar todos os óbitos naturais, fossem cardíacos ou não. Para a mortalidade total, foram considerados os óbitos intra-hospitalares por infecção relacionada a DCEI e os óbitos durante o seguimento clínico após a alta hospitalar.

### Análise Estatística

Para a análise dos dados, foi utilizado o programa SPSS, versão 14.0. As variáveis categóricas foram expressas em números e proporção, e as variáveis contínuas foram expressas em médias e desvio padrão. Os testes do qui-quadrado e de Fisher, quando apropriados, foram utilizados para estudar associações entre variáveis categóricas. Para a comparação entre variáveis contínuas, foi utilizado o teste t de Student não pareado, em razão da distribuição normal por meio do teste de Kolmogorov-Smirnov. O intervalo de confiança (IC) foi de 95%. Foi realizada análise da sobrevida por meio da curva de Kaplan-Meier. Foi aplicado o teste de *log-rank* para comparar as curvas de sobrevida. O nível de significância adotado na análise estatística foi de 5%.

## Resultados

### Características Gerais da Casuística

**Figura 1:**
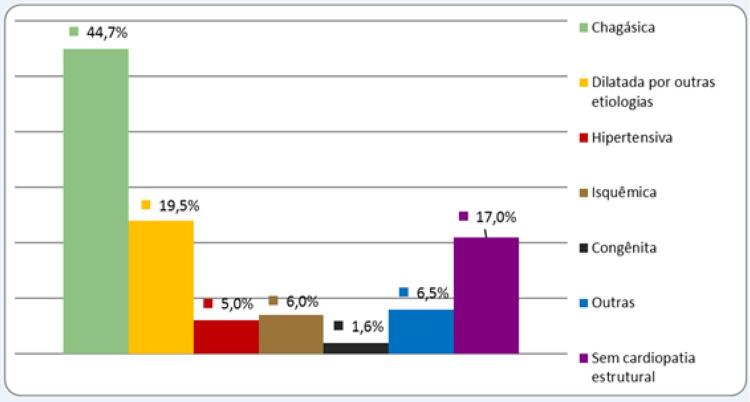
– Principais cardiopatias de base.

A média de idade dos 123 pacientes com infecção relacionada a DCEI foi de 60,1±19,4 anos (variando de 3 meses a 97 anos), sendo 71 (57,7%) do sexo masculino. O número médio de procedimentos realizados, considerando implantes, trocas e manipulações de eletrodos, foi de 1,7. A média da fração de ejeção do ventrículo esquerdo (FEVE) foi de 48,4%. Considerando o tempo de inclusão dos pacientes com infecção relacionada a DCEI, o qual foi de 16 anos, a taxa anual de infecção foi de 1,2 por 1.000 procedimentos. As principais cardiopatias de base são apresentadas na Figura 1. Em relação aos DCEI, o modo de estimulação foi MP em modo VVI em 38,2%, em modo DDD em 30,9%, em modo AAI em 2,4%, CDI em 19,5% e terapia de ressincronização cardíaca (TRC) em 9% dos pacientes.

### Variáveis Relacionadas à Infecção

Todos os pacientes apresentaram sinais e/ou sintomas sugestivos de infecção por DCEI. Foram observados secreção em bolsa em 39 (31,7%) pacientes, febre e mal-estar generalizado em 23 (18,6%) e bolsa com sinais de hiperemia e flutuação em 16 (13,0%). Houve extrusão do gerador em 45 (36,5%) pacientes.

Foram colhidas hemoculturas de todos os pacientes. O agente etiológico mais prevalente foi o estafilococo, isolado em culturas de 63 (51,2%) pacientes, seguido por estreptococo, encontrado em 2 (1,6%). Outros agentes, como *serratia, pseudomnas aeruginosa, enterococcus faecalis* e *klebsiella*, foram isolados em 20 (16,3%) pacientes. Houve presença de mais de um agente etiológico em 36 (29,7%) hemoculturas, e 38 (30,9%) foram negativas.

Foram realizadas culturas da secreção da loja do gerador e da ponta de eletrodos em 74 pacientes. Entre os resultados da primeira, foram encontrados os agentes etiológicos *S. aureus* e *S. epidermidis* em 15 (20,2%) e 5 (6,7%) pacientes, respectivamente. Os demais agentes etiológicos, como *pseudomonas, escherichia coli* e *acinetobacter baumannii*, foram isolados em 8 (10,8%) amostras. A cultura de ponta de cateter identificou os agentes etiológicos *S. aureus* e *S. epidermidis* em 21 (28,3%) e 18 (24,3%) pacientes, respectivamente. Os demais agentes, como *serratia marcescens, pseudomonas* e *aeromonas hydrophila*, foram isolados em 7 (9,4%) amostras.

O ecocardiograma transesofágico foi realizado em 91 (73,9%) pacientes, sendo que 44 (35,7%) apresentaram imagem sugestiva de vegetação, enquanto o ecocardiograma transtorácico foi utilizado em 114 pacientes. Outros dados laboratoriais, como leucócitos, proteína C-reativa, intervalo de tempo entre o último implante e o diagnóstico de infecção e o tempo de internação, estão dispostos na Tabela 1.

**Tabela 1:** – Variáveis relacionadas à infecção

Variáveis	Média	Desvio padrão	Mínimo	Máximo	Mediana
∆ tempo (dias)	563,36	936,43	1	5895	138,5
Leucócitos (ml/mm^3^)	9.502,7	5.900,9	1.008,0	51.310,0	8.350,0
PCR (mg/L)	68,7	81,3	3	376,6	34,3
Tempo de internação (dias)	35,3	22,3	1	131	29,0

*tempo: intervalo de tempo entre o último implante e o diagnóstico de infecção; PCR: proteína C-reativa.*

Ocorreu infecção relacionada a DCEI causada pela realização do primeiro implante em 58 (47,1%) pacientes. Em 55 (44,7%), ocorreu devido à troca de gerador e, em 10 (8,1%), por manipulações como plastia de loja de gerador, *upgrade* e reposicionamento de eletrodo. Houve infecção precoce (considerando o intervalo de tempo entre o procedimento e o diagnóstico de infecção inferior a 1 ano) em 78 (63,4%) pacientes. Não houve influência das variáveis sexo, idade, índice de massa corporal, número de procedimentos, tipo de dispositivo e fração de ejeção.

### Abordagens Farmacológica e Não Farmacológica em Relação à Infecção

O antibiótico mais utilizado foi a vancomicina [91 (73,9%) pacientes], seguido pela oxacilina [20 (16,2%) pacientes]. A retirada total do sistema foi feita em 105 (85,4%) pacientes, enquanto a parcial foi feita em 11 (8,9%). Foram tratados somente com antibióticos sete (5,7%) pacientes. Entre aqueles que foram submetidos à retirada parcial, oito (6,5%) apresentaram recidiva da infecção.

Foram submetidos a reimplante de novos sistemas 108 pacientes, sendo por via endocárdica em 64 (52%) e por via epicárdica em 44 (35,7%). O reimplante do DCEI não foi realizado em 15 pacientes (12,1%) pelos seguintes motivos: quatro foram submetidos a transplante cardíaco, três faleceram antes do procedimento e a família de um dos pacientes não autorizou o reimplante. Em três pacientes, a equipe médica optou por não realizar o procedimento.

### Evolução Intra-hospitalar dos Pacientes

A média do período de internação hospitalar foi de 35,3±22,3 dias, variando de 1 a 131 dias. Houve evolução sem complicações no período da internação em 40 (32,5%) pacientes. Houve piora da função renal em 37 (30,0%) pacientes; tromboembolismo pulmonar, encefalopatia e meningite em 27 (21,9%); derrame pleural em 11 (8,9%); e necessidade de ventilação mecânica em oito (6,5%). Ainda, 71 (57,7%) pacientes evoluíram com endocardite infecciosa e, entre esses, 19 (15,4%) evoluíram com sepse. Foi diagnosticada sepse em 23 (18,7%) pacientes, sendo que 15 (12,1%) faleceram em virtude disso. Em relação à endocardite e ao tipo de dispositivo, 55,6% dos pacientes que cursaram com endocardite estavam com MP, 62,5% com CDI e 54,5% com TRC (p =0,65). Os demais dados sobre as variáveis associadas ou não a endocardite infecciosa estão dispostos na Tabela 2.

**Tabela 2:** – Comparação das médias das variáveis entre os grupos de pacientes com e sem endocardite infecciosa

Variáveis	Grupo sem endocardite	Grupo com endocardite	Valor-p*
Sexo masculino	31 (25,2%)	39 (31,7%)	0,51
Idade (anos)	60,2±18,9	60,0±19,9	0,95
IMC (kg/m2)	24,5±5,1	24,2±4,9	0,77
Fração de ejeção (%)	45,0±16,4	50,4±17,7	0,99
Extrusão de gerador	24 (19,5%)	21 (17,0%)	0,045
N de procedimentos	1,6±0,8	1,8±0,9	0,405
Sepse	4 (3,2%)	19 (15,4%)	0,010
Leucócitos (ml/mm^3^)	8.638±9.886	8.568±7.351	0,96
PCR (mg/L)	51,6±56,4	80,9±93,6	0,043

IMC: índice de massa corpórea; PCR: proteína C-reativa; N: número. *Teste qui-quadrado ou Fisher ou teste t de Student não pareado.

A mortalidade intra-hospitalar foi de 19,5% (24 pacientes); todos os óbitos foram por infecção relacionada a DCEI. A comparação entre os pacientes que cursaram sem e com morte intra-hospitalar é apresentada na Tabela 3. O risco para morte intra-hospitalar em relação ao curso com endocardite infecciosa foi de 4,47 (com IC95% entre 1,42 e 14,1). Quanto ao curso com sepse, o risco para morte intra-hospitalar foi de 4,1 (IC95% entre 1,3 e 12,9). Em relação ao tipo de dispositivo, 18 (20,5%) pacientes com MP, 4 com CDI (16,6%) e 2 com TRC (18,2%) faleceram no período intra-hospitalar (p = 0,42).

**Tabela 3:** – Análise entre os pacientes que cursaram sem e com morte intra-hospitar

Variáveis	Grupo sem morte intra-hospitalar (n=99)	Grupo com morte intra-hospitalar (n=24)	Valor-p*
Sexo masculino	57 (46,4 %)	14 (11,3%)	0,94
Idade (anos)	59,9±18,6	61,2±22,8	0,79
IMC (kg/m^2^)	24,7±4,9	22,9±5,9	0,21
Fração de ejeção (%)	49,0±17,3	45,9±17,9	0,45
N de procedimentos prévios	1,73±0,9	1,95±0,9	0,317
Proporção de pacientes que cursaram com EI	52,0	83,3	0,005
Proporção de pacientes que cursaram com sepse	8,1	62,5	< 0,0001
Leucócitos (ml/mm^3^)	8.580±8.646	8.661±7.777	0,96
PCR (mg/L)	62,73±72,0	94,76±111,4	0,22

EI: endocardite infecciosa; IMC: índice de massa corporal; N: número; PCR: proteína C-reativa. *Teste qui-quadrado ou Fisher ou teste t de Student não pareado.

### Evolução Após a Alta Hospitalar

Houve mais de uma infecção em oito pacientes. Foi dada alta hospitalar a 99 (80,4%) pacientes, com seguimento clínico médio de 43,8 meses e mediana de 28,3, variando de 0,6 a 177 meses. A taxa de mortalidade após a alta hospitalar foi de 29,3% (29 pacientes), ocorrendo entre 3,94 a 164,5 meses.

### Curvas de Sobrevida

Utilizando a curva de Kaplan-Meier e considerando como base prognóstica a ocorrência de morte total (por causa cardíaca e não cardíaca), foram construídas curvas de sobrevida. O teste de *log-rank* (Mantel-Cox) foi aplicado para comparar as curvas.

#### Sobrevida Total

**Figura 2:**
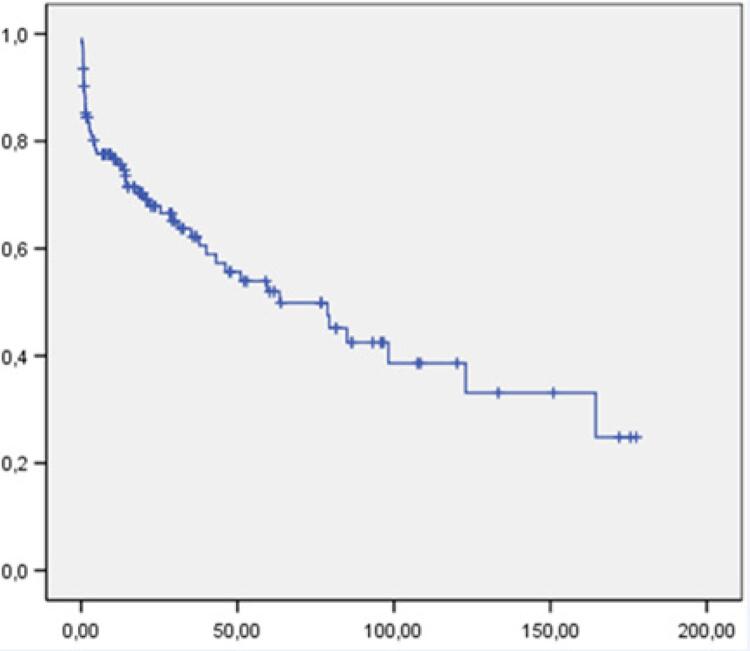
– Curva de sobrevida de toda a casuística. %”Eixo horizontal: tempo em meses; eixo vertical: probabilidade acumulada de sobrevida %.

Durante todo o seguimento clínico de 43,8 meses, 53 (43,0%) pacientes faleceram, sendo 24 no período intra-hopitalar e 29 após a alta hospitalar. A taxa anual de mortalidade total foi 11,8% e 0,52 por 1.000 procedimentos/ano. A curva de sobrevida total dessa casuística é apresentada na Figura 2.

#### Sepse

**Figura 3:**
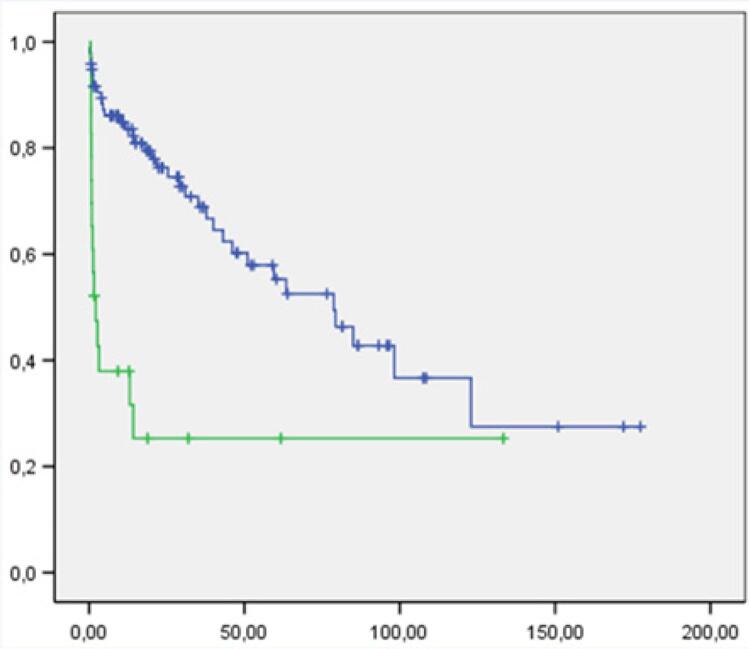
– Curva de sobrevida em relação à sepse. Eixo horizontal: tempo em meses; eixo vertical: probabilidade acumulada de sobrevida. Curva azul: pacientes que cursaram sem sepse durante a internação; curva verde: pacientes que cursaram com sepse.

Dos 23 pacientes diagnosticados com sepse, 15 (65,2%) faleceram durante o seguimento de 43,8 meses, com p < 0,0001 pelo teste de *log-rank* (Figura 3). A análise com seguimento de 6 e 36 meses apresentou o mesmo valor-p.

#### Outras Variáveis

Não houve diferença na sobrevida quanto ao sexo (p = 0,89) e ao agente etiológico (p = 0,11). Em relação ao tipo de dispositivo, a taxa de mortalidade foi de 48,8% entre os pacientes com MP, 29,2% entre os pacientes com CDI e 27,2% entre aqueles com TRC (p = 0,92). Entre os pacientes que apresentaram endocardite durante a internação, 47,8% faleceram durante o seguimento clínico de 43,8 meses (p = 0,93), sem diferença significativa, inclusive com seguimento de 6 e 36 meses (p = 0,11 e 0,08, respectivamente). Considerando a fração de ejeção < 50% e ≥ 50%, a taxa de mortalidade foi de 44,2% e 41,5%, respectivamente, durante todo o seguimento clínico (p = 0,06). Quanto à modalidade de tratamento, faleceram 42,8% dos pacientes tratados somente com antibioticoterapia, 18,2% dos submetidos à retirada parcial do sistema e 47,7% dos submetidos à retirada total do sistema (p = 0,07). Tomando por base prognóstica o tipo de reimplante, 32,8% dos pacientes submetidos ao implante endocárdico e 52,2% daqueles submetidos ao epicárdico faleceram (p = 0,04).

## Comparação entre Chagásicos e Não Chagásicos

Na comparação entre pacientes chagásicos e não chagásicos, não houve diferença quanto às variáveis (idade, sexo, tipo de dispositivo, número de procedimentos, intervalo entre o último implante e o diagnóstico de infecção, valores de leucócitos, proteína C-reativa, FEVE, tempo de internação, agente infeccioso, presença de extrusão do gerador, proporção de endocardite infecciosa e de sepse e modalidade de tratamento) nem quanto à mortalidade (intra-hospitalar e após a alta).

## Discussão

O implante de DCEI aumentou de forma significativa ao longo dos últimos anos devido à maior abrangência das indicações desses dispositivos e em virtude do aumento da expectativa de vida e do maior número de cardiopatas. A infecção relacionada a DCEI constitui um grave problema, com elevados índices de morbidade e mortalidade e com grande impacto socioeconômico em razão de seu alto custo de tratamento.^7,18,19^ No presente estudo, a média da idade dos pacientes foi semelhante à observada em outros estudos,^9,13^ assim como o predomínio do sexo masculino entre os pacientes com infecção relacionada a DCEI.^9,20,21^A etiologia principal da cardiopatia de base no presente estudo, a chagásica, foi distinta da de outros países, nos quais há prevalência da cardiopatia isquêmica.^4,9^

As taxas de infecção podem variar dependendo da duração do seguimento e do tipo de dispositivo e de procedimento.^5,21^ Este estudo revelou que houve maior proporção de infecção relacionada a procedimentos de troca de gerador, *upgrade* e plastia de bolsa. O tempo entre a última manipulação e o diagnóstico de infecção na literatura foi, em média, de 20 meses,^13^ semelhante ao presente estudo, mas pode variar dependendo do dispositivo, com intervalo de 4,2 meses em relação ao CDI.^22^ Quanto à etiologia da infecção, as espécies de estafilococos causam a maior parte das infecções relacionadas a DCEI, sendo responsáveis por 60% a 80% dos casos relatados,^1,23^ taxa maior que a do presente estudo. Contudo, houve alta taxa de hemoculturas negativas, diferindo do reportado na literatura.^1,5,24^ Essa discordância pode ser atribuída ao uso prévio de antibióticos pelos pacientes, antes de seu ingresso no hospital.^25^

Para o diagnóstico da infecção relacionada com DCEI, além do método clínico, estão indicados exames laboratoriais e ecocardiograma. O ecocardiograma transesofágico é o mais indicado para o diagnóstico de infecção endovascular em razão de sua sensibilidade de 88% e especificidade de 99%. Já o ecocardiograma transtorácico apresenta sensibilidade de apenas 32%.^3^ Portanto, apesar da alta sensibilidade do exame e de sua indicação precisa nesse quadro, a correlação clínica e os resultados de hemoculturas são fundamentais para o diagnóstico e para as complicações referentes à essa infecção.

Uma dessas complicações é a endocardite, infecção grave que pode ocorrer entre 0,06 a 7,0% dos casos de infecção relacionada a DCEI.^13^ Apresenta incidência anual de 1,83 casos/milhão de habitantes e 390 casos/milhão de portadores de MP^26^ e mortalidade relatada de até 26%.^27^ Na população estudada, 57,7% dos pacientes desenvolveram endocardite. Em concordância com a literatura, o presente estudo mostrou o agravamento do prognóstico daqueles que cursaram com endocardite. Outra complicação foi a sepse, também contribuindo para o elevado número de óbitos, cuja taxa, segundo a literatura, pode variar de 32,2 a 51,1%, tendo como principal agente o *S. aureus*.^28,29^A tendência de associação inversa entre endocardite e extrusão do gerador no presente estudo pode ser explicada pelo número de pacientes com endocardite e extrusão, resultando em um viés de confusão, uma vez que o sinal extrusão pode ou não estar presente nos casos de endocardite.

Estudos recomendam o uso da vancomicina como prioritária no início da terapia antibiótica empírica no tratamento da infecção relacionada a DCEI, até que sejam verificados os resultados das hemoculturas.^1^Em concordância com a literatura, no estudo em questão, a vancomicina foi utilizada em 73,9% dos casos. Além da terapia com antibióticos, há outras modalidades adicionais de tratamento, como a retirada precoce e total do sistema do dispositivo com impacto favorável na evolução dos pacientes, associada a melhor sobrevida.^11^ Foram observados, no presente estudo, os benefícios da realização da retirada total do sistema de DCEI, com o objetivo de cura da infecção sem recidiva. Entretanto, a retirada total do sistema envolve, às vezes, cirurgia mais complexa como a cardiotomia, que pode agravar o quadro dos pacientes em questão. Dados da literatura demonstraram que a rápida extração do dispositivo e de seus eletrodos, associada a uma antibioticoterapia adequada com reimplante de novo dispositivo epicárdico ou contralateral, resultou em elevado índice de cura, com baixo risco de mortalidade operatória e infecção recorrente.^30^ A técnica percutânea de extração de eletrodos apresenta menor taxa de risco, entretanto, a mortalidade pode atingir 1,2% em centros experientes devido a sangramentos, perfurações vasculares e tamponamento cardíaco.^31^

A infecção relacionada a DCEI pode resultar em um tempo prolongado de internação, o qual é estendido por mais de 13% em relação ao período de internação para o implante de novo dispositivo.^32^O tratamento com antibióticos, o procedimento de extração e de reimplante e as complicações associadas contribuem para esse aumento no tempo de internação, com impacto, também, econômico. O tempo médio de internação no presente estudo foi de 35,5 dias, enquanto nos relatos da literatura foi de 17 dias.^22^ Essa diferença pode ser explicada pela maior proporção de pacientes com endocardite infecciosa neste estudo, resultando em tratamento mais extenso com antibióticos, conforme preconizado pela literatura.^3,15^

Além da morbidade, a infecção relacionada a DCEI também apresenta mortalidade tanto intra-hospitalar quanto pós-alta hospitalar. A mortalidade intra-hospitalar demonstrou uma variação ampla, segundo a literatura, dependendo do número de pacientes, da idade avançada e da presença de comorbidades e de complicações durante o tratamento, com taxas entre 6 e 14%. Além disso, apresentou mortalidade total de aproximadamente 20% em um ano,^1,6,9^ atingindo 26,9% durante o seguimento de 5 anos.^1,8,33^ Na população estudada, a taxa de mortalidade intra-hospitalar foi maior do que as demostradas nos estudos citados, o que pode ser justificado pelo maior número de pacientes que desenvolveram endocardite e sepse. Em relação ao período após a alta hospitalar, estudos com seguimento de até 2 anos evidenciaram que a taxa de total de mortes pode ser considerável e variar de 6% a 35%.^34,35^No presente estudo, a taxa de mortalidade após a alta hospitalar foi de 23,5% durante o seguimento de 43,8 meses, com taxa anual de 14,5%, dentro da faixa de taxas descritas na literatura.

Como já descrito previamente, há variáveis associadas a desfechos desfavoráveis e preditores de mortalidade.^1,10-13,21^No estudo em questão, não houve associação significativa, pelas curvas de Kaplan-Meier, entre a sobrevida e o tipo de dispositivo, o curso de endocardite infecciosa durante a internação e a modalidade de tratamento. Entretanto, houve diferença significativa em relação à complicação de sepse durante a internação, com menor sobrevida após a alta, assim como entre aqueles submetidos ao implante epicárdico.

Em relação à modalidade de tratamento, Kim et al.,^9^relataram que pacientes tratados de forma conservadora, ou seja, somente com antibioticoterapia, apresentaram taxa de óbito elevada em tempo com média de 25 dias.^9^Também há estudos relatando que a remoção precoce do dispositivo esteve associada a maior sobrevida dos pacientes.^2,36^ Quando não há remoção total do dispositivo, a mortalidade pode aumentar em 7 vezes dentro de 30 dias.^3^ Uma publicação atual, com 6.859 pacientes sem infecção relacionada a DCEI, comparando a evolução entre aqueles submetidos à extração e aqueles com abandono dos eletrodos, demonstrou que a remoção dos eletrodos foi associada a menor taxa de infecção durante o período de 5 anos, porém não houve impacto sobre a sobrevida dos pacientes.^37^ De maneira correlata, porém com população incluindo pacientes com infecção relacionada a DCEI, um estudo de caso-controle demonstrou taxas similares de mortalidade em pacientes com e sem infecção.^34^ Isso reflete a heterogeneidade das casuísticas dos estudos quanto ao perfil clínico, tempo de diagnóstico e de intervenção e quanto às comorbidades, variáveis que interferem, também, na sobrevida. Além disso, uma pesquisa publicada em junho de 2019, com a participação de 62 países, demonstrou que somente 39,9% dos profissionais que realizam o implante de DCEI fazem irrigação da bolsa com antibiótico e somente 44% administram antibiótico profilático, com remoção completa do sistema em 62% das vezes em caso de infecção,^38^ o que ilustra a disparidade de abordagem em pacientes com infecção relacionadas ao DCEI.

Quanto ao implante epicárdico, um estudo comparando o reimplante de eletrodos de MP após a infecção demonstrou que os 65 pacientes submetidos a acesso epicárdico apresentaram um risco de endocardite tardia ou reintervenção do dispositivo 3,6 vezes maior do que os 37 pacientes submetidos a MP temporário e posterior reimplante endocárdico.^39^ Isso foi explicado pelas complicações associadas ao reimplante epicárdico.

A etiologia da cardiopatia de pacientes com DCEI influencia sua evolução. O prognóstico de pacientes com cardiopatia chagásica crônica é desfavorável quando comparado ao de outras etiologias.^40^ Na literatura, não há estudos específicos sobre infecção relacionada a DCEI e etiologia chagásica, exceto um estudo sobre o diagnóstico microbiano com a cultura de fluidos.^41^ Comparando 15 pacientes com infecção e 68 sem infecção relacionada a DCEI, com um total de 19 chagásicos, não houve diferença entre os grupos quanto a essa etiologia. No presente estudo, com um total de 55 chagásicos, a comparação entre pacientes chagásicos e não chagásicos não demonstrou diferença entre as variáveis e quanto à evolução.

### Limitações do Estudo

A parte retrospectiva do estudo foi uma desvantagem devido à menor disponibilidade de registros adequados nos prontuários, além da subnotificação de pacientes com infecção relacionada a DCEI. Isso pode ter repercutido na taxa de infecção, com algum viés na análise. Além disso, em virtude do longo tempo de inclusão dos pacientes, as técnicas e os aparelhos de ecocardiograma foram distintos durante o período, sem uniformidade de sua realização, impedindo a verificação da valva acometida nos casos de endocardite infecciosa. Como nem todos os pacientes foram submetidos ao ecocardiograma transesofágico, a taxa de endocardite pode ter sido subestimada.

## Conclusões

A taxa de infecção foi de 1,9% (1,2 por 1.000 procedimentos/ano), com predomínio em homens e em pacientes com miocardiopatia dilatada. Durante a internação, a incidência de endocardite infecciosa foi de 57,7% e a de sepse, 18,7%. Foi feita remoção total do sistema na maioria dos pacientes (85,4%). A taxa de mortalidade intra-hospitalar foi de 19,5% e houve associação com endocardite e sepse. Após a alta hospitalar, a taxa de mortalidade anual foi de 11,8%, com influência somente da ocorrência de sepse durante a internação e do implante epicárdico.
