# Supplementary material for: Perfil Clínico e Evolução de Pacientes com Infecção Relacionada a Dispositivos Cardíacos Eletrônicos Implantáveis
Source: Arq Bras Cardiol. 2021 Jun 8;116(6):1080–8. [Article in Portuguese] doi: 10.36660/abc.20190546 (PMC8288527; doi:10.36660/abc.20190546)
Supplement: Translation

# Translation: Clinical Profile and Outcome of Patients with Cardiac Implantable Electronic Device-Related Infection

**Authors:** Alessandra de Souza Maciel, Rose Mary Ferreira Lisboa da Silva

**Affiliations:** 1 Brazil Universidade Federal de Minas Gerais - Hospital das Clínicas, Belo Horizonte, MG - Brazil; 2 Brazil Universidade Federal de Minas Gerais - Departamento de Clínica Médica, Belo Horizonte, MG - Brazil

**Keywords:** Cardiac Pacemaker Implantation, Cardiovascular Surgical Procedures, Bacteria, Endocarditis, Outcome, infection

## Abstract

**Background:**

In recent years, the incidence of infections related to cardiac implantable electronic devices (CIED) has increased sharply, impacting mortality.

**Objective:**

To verify the proportion of patients with CIED infection; to analyze their clinical profile and the variables related to the infection and its progression.

**Methods:**

Retrospective and longitudinal observational study including 123 patients with CIED infection among 6406 procedures. Parametric tests and a level of significance of 5% were used in the statistical analyses

**Results:**

The mean age of patients was 60.1 years and mean length of stay in hospital was 35.3 days; most (71) patients were male, and the system was completely removed in 105 cases. Infectious endocarditis (IE) and sepsis were observed in 71 and 23 patients, respectively. Intra-hospital mortality was 19.5%. IE was associated with extrusion of the generator (17.0% vs 19.5% with and without IE, respectively, p = 0.04, inverse association) and sepsis (15.4% vs 3.2%, p = 0.01). Intra-hospital death was associated with IE (83.3% vs 52.0% with and without intra-hospital death, respectively, p = 0.005) and sepsis (62.5% vs 8.1%, p < 0.0001). Ninety-nine patients were discharged. During a mean follow-up of 43.8 months, mortality rate was 43%; among patients with sepsis, it was 65.2% (p < 0.0001). By applying a Kaplan-Meier survival curve, we did not indicate significant associations with sex, etiologic agent, ejection fraction, IE, or treatment modality. The death rate was 32.8% for patients subjected to endocardial electrode reimplantation and 52.2% for epicardial reimplantation (p = 0.04). Chagasic etiology (44.7% of the baseline heart diseases) did not influence clinical and laboratory variables or disease progression.

**Conclusion:**

The infection rate was 1.9%, mostly in men. We observed an association of intra-hospital mortality with IE and sepsis. After discharge, the annual mortality rate was 11.8%, influenced by sepsis during hospitalization and epicardial implantation. (Arq Bras Cardiol. 2021; [online].ahead print, PP.0-0)

## Introduction

The use of cardiac implantable electronic devices (CIED) has grown exponentially in the las 10 years owing to technology advancements, broadening indications, and increasing life expectancy. On the other hand, during this period, an important and disproportionate 210% increase in the incidence of CIED-related infections has brought this number to 19.9%.^1-4^ These infections are related to the type of device and number of interventions.^5^ After device replacement, the risk of infection is around 5%, which indicates a 2–4-fold increased risk when compared to a primary implant.^5,6^Other factors are also associated with increased infection rates, such as sex, age, comorbidities, and lack of prophylaxis.^7,8^

This type of infection causes significant morbidity, and intra-hospital mortality varies from 6% to 14% with a total mortality of approximately 20% in one year.^1,6,9^ Some variables are also associated with unfavorable outcomes and mortality predictors, such as the patient’s age, use of temporary pacemaker (PM), device replacements, *Staphylococcus* sp. as the etiologic agent, prosthetic heart valves, time to device removal, kidney disease, need for blood transfusion, and endocarditis.^1,10-13^ The risk of death due to CIED-related infection depends on the device type and persists with time. The 20% mortality rate continues for 3 years for single- or dual-chamber PMs, and for 2 years for the implantable cardioverter-defibrillator (ICD).^14^

In Brazil, information on this subject is scarce; moreover, patient characteristics and etiologies for CIED implantation are different from those observed in developed countries. Therefore, recognizing these patients’ profile and their clinical course is an important initial step for implementing the guidelines established by the literature.^15^ In view of this information, the objectives of this study were to verify the proportion of patients with CIED-related infection and to analyze their clinical and laboratory profiles, variables related to the infection, and its progression.

## Methods

This is a retrospective and longitudinal observational cohort study. Our population consisted of 123 patients with device-related infection, of both sexes and all ages, selected among 6406 CIED implantation procedures performed between 2001 and 2017. Patients with DCEI infection but who underwent implantation of the device in other hospitals were also excluded. We excluded patients with infections related to temporary PMs. Both the research project and the free and informed consent form were approved by the institution’s Ethics and Research Committee according to Resolution No. 466/2012. We analyzed clinical and laboratory variables, as well as pharmacological and non-pharmacological treatment data. The diagnosis of CIED-related infection considered clinical examinations associated with a complete blood count, C-reactive protein, blood cultures, and echocardiogram examinations. Infectious endocarditis was diagnosed using modified Duke criteria.^16^

In the institution where the study was conducted, prophylaxis and treatment of CIED-related infections included aseptic techniques with chlorhexidine detergent showers the night before and in the morning of the procedure, hair removal, surgical degerming, and skin antisepsis with chlorhexidine detergent for 2 min, removing excess product and applying an alcoholic chlorhexidine solution. According to the same protocol, antibiotic prophylaxis was performed 1 h before the procedure with a single dose of 2g of cefazolin.

Sepsis was defined as a potentially fatal organic dysfunction caused by a dysregulated immune response to infection.^17^ Intra-hospital mortality considered deaths due to infection during hospitalization. After discharge, surviving patients were followed-up for a minimum period of 6 months. We considered post-discharge deaths as natural deaths of cardiac or non-cardiac causes. Total mortality considered intra-hospital deaths (due to CIED-related infection) and post-discharge deaths during follow-up.

### Statistical Analysis

Data were analyzed using SPSS version 14.0. Results were expressed as absolute numbers and proportions for categorical variables, and as means and standard deviations for continuous variables. When appropriate, chi-squared and Fisher’s tests were used for verifying associations between categorical variables. For comparing continuous variables, an unpaired Student’s t-test was used after verifying a normal distribution through the Kolmogorov-Smirnov test. The confidence interval used in the analyses was 95%. Survival analysis used Kaplan-Meier curves, which were compared using a log-rank test. The level of significance used in the analyses was 5%.

## Results

### General Characteristics of the Studied Cases

The mean age of the 123 patients with CIED-related infection was 60.1 ± 19.4 years (ranging from 3 months to 97 years); 71 (57.7%) patients were male. The mean number of procedures considering implantations, replacements, and electrode manipulations was 1.7. Mean left ventricular ejection fraction was 48.4%. Considering the period of patient inclusion (16 years), the annual infection rate was 1.2 per 1000 procedures.

The main baseline heart diseases are displayed in Figure 1. Regarding CIED, stimulation modes were: PM VVI mode in 38.2% of the patients, DDD mode in 30.9%, and AAI mode in 2.4% of the patients; ICD in 19.5% of the patients; and cardiac resynchronization therapy (CRT) in 9% of patients.

**Figure 1:** – Main baseline heart diseases.

### Variables Related to Infection

All patients presented signs and/or symptoms suggestive of CIED-related infection. We observed pocket discharge in 39 (31.7%) patients, fever and malaise in 23 (18.6%) patients, and pocket with signs of hyperemia and fluctuation in 16 (13.0%) patients. Forty-five (36.5%) patients presented extrusion of the generator.

We performed blood cultures with samples from all patients. The most prevalent etiologic agent, isolated in the cultures of 63 (51.2%) patients, was *Staphylococcus aureus,* followed by *Streptococcus* epidermidis in 2 (1.6%) patients. Other agents such as Serratia sp., *Pseudomonas aeruginosa, Enterococcus faecalis*, and *Klebsiella* sp. were isolated in 20 (16.3%) patients. Thirty-six (29.7%) patients presented more than one etiologic agent. Blood cultures were negative in 38 (30.9%) patients.

Seventy-four patients had blood cultures performed with samples collected from the generator pocket and electrode tips. In cultures performed with generator pocket samples, *S. aureus* was found in 15 (20.2%) patients, and *S. epidermidis* was found in 5 (6.7%) patients. Other etiologic agents such as *Pseudomonas* sp., *Escherichia coli*, and *Acinetobacter baumannii* were isolated in 8 (10.8%) samples.

Catheter tip cultures demonstrated *S. aureus* as etiologic agent in 21 (28.3%) patients. Other agents such as *Serratia marcescens, Pseudomonas* sp., and *Aeromonas hydrophila* were isolated in 7 (9.4%) samples.

One hundred and fourteen patients underwent transthoracic echocardiography. Transesophageal echocardiography was performed in 91 (73.9%) patients, of which 44 (35.7%) yielded images suggestive of vegetation.

Other laboratory data (leucocytes, C-reactive protein) and the time between the last implantation and the diagnosis of infection, as well as length of stay, are shown in Table 1.

**Table 1:** – Variables related to the infection

Variables	Mean	Standard deviation	Minimum	Maximum	Median
∆ time (days)	563.36	936.43	1	5895	138.5
Leukocytes (cells/mm^3^)	9502.7	5900.9	1008.0	51310.0	8350.0
C-reactive protein (mg/L)	68.7	81.3	3	376.6	34.3
Length of stay (days)	35.3	22.3	1	131	29.0

*∆ time: time between the last implantation and the diagnosis of infection.*

CIED-related infection occurred after the first implantation procedure in 58 (47.1%) patients. In 55 (44.7%) patients, it happened at generator replacement, and in 10 (8.1%) patients, it occurred after device manipulations such as generator pocket revision, device upgrade, and electrode repositioning.

We observed early infections (when the time between the procedure and diagnosis of infection was shorter than a year) in 78 (63.4%) patients. Sex, age, body mass index, number of procedures, device type, and ejection fraction did not influence the occurrence of infection.

### Pharmacological and Non-pharmacological Approaches to Infection

The most widely used antibiotic was vancomycin, in 91 (73.9%) patients, followed by oxacillin in 20 (16.2%) patients. The system was totally removed in 105 (85.4%) patients and was partially removed in 11 (8.9%) cases. Seven (5.7%) patients were treated only with antibiotics. Among those who underwent partial removal, 8 (6.5%) had infection relapse.

New systems were reimplanted in 108 patients, of which 64 (52%) underwent endocardial reimplantation and 44 (35.7%), epicardial reimplantation. Fifteen (12.1%) patients did not undergo CIED reimplantation due to the following reasons: 4 were subjected to cardiac transplants, 3 patients died before reimplantation, and 1 patient’s family did not provide authorization for reimplantation. In 3 cases, the medical team opted not to perform reimplantation.

### Intra-hospital Patient Course

Mean length of stay was 35.3 ± 22.3 days, ranging from 1 to 131 days. Forty (32.5%) patients progressed without complications during hospitalization. Thirty-seven (30.0%) patients had worsening renal function, 27 (21.9%) had pulmonary thromboembolism, encephalopathy, and meningitis, 11 (8.9%) had pleural effusion, and 8 (6.5%) needed mechanical ventilation. Seventy-one (57.7%) patients had infectious endocarditis, of which 19 (15.4%) progressed to sepsis. Sepsis was diagnosed in 23 (18.7%) patients, and 15 (12.1%) died due to this condition. As for endocarditis and device types, 55.6% of the patients who had endocarditis had a PM, while 62.5% had an ICD, and 54.5% had a CRT device (p = 0.65). Other data on variables associated (or not) to infectious endocarditis are shown in Table 2.

**Table 2:** – Comparison between variable means in patients with or without infectious endocarditis

Variables	No endocarditis	Endocarditis	p-value*
Male sex	31 (25.2%)	39 (31.7%)	0.51
Age (years)	60.2 ± 18.9	60.0 ± 19.9	0.95
BMI (kg/m^2^)	24.5 ± 5.1	24.2 ± 4.9	0.77
Ejection fraction (%)	45.0 ± 16.4	50.4 ± 17.7	0.99
Extrusion of the generator	24 (19.5%)	21 (17.0%)	0.045
No. of procedures	1.6 ± 0.8	1.8 ± 0.9	0.405
Sepsis	4 (3.2%)	19 (15.4%)	0.010
Leukocytes (cells/mm^3^)	8638 ± 9886	8568 ± 7351	0.96
C-reactive protein (mg/L)	51.6 ± 56.4	80.9 ± 93.6	0.043

BMI: body mass index. * chi-squared or Fisher’s tests, or unpaired Student’s t-test.

Intra-hospital mortality was 19.5% (24 patients); all deaths were due to CIED-related infection. A comparison between patients who progressed or not to intra-hospital death is presented in 3.

The risk of intra-hospital death, regarding a clinical course with infectious endocarditis, was 4.47 (95% confidence interval 1.42–14.1). As for sepsis, this risk was 4.1 (95% confidence interval 1.3–12.9).

According to device type, 18 (20.5%) patients with PM, 4 (16.6%) with ICD, and 2 (18.2%) with CRT devices died in the hospital (p = 0.42).

**Table 3:** – Analysis between patients who progressed or not to intra-hospital death

Variables	Intra-hospital death (n = 99)	Intra-hospital death (n = 24)	p-value*
Male sex	57 (46.4 %)	14 (11.3%)	0.94
Age (years)	59.9 ± 18.6	61.2 ± 22.8	0.79
BMI (kg/m^2^)	24.7 ± 4.9	22.9 ± 5.9	0.21
Ejection fraction (%)	49.0 ± 17.3	45.9 ± 17.9	0.45
No. of previous procedures	1.73 ± 0.9	1.95 ± 0.9	0.317
Proportion of patients who progressed to IE	52.0	83.3	0.005
Proportion of patients who progressed to sepsis	8.1	62.5	< 0.0001
Leukocytes (cells/mm^3^)	8580 ± 8646	8661 ± 7777	0.96
C-reactive protein (mg/L)	62.73 ± 72.0	94.76 ± 111.4	0.22

BMI: body mass index; IE: infectious endocarditis. * chi-squared or Fisher’s tests, or unpaired Student’s t-test.

### Progression After Hospital Discharge

Eight patients presented more than one infection. Ninety-nine (80.4%) patients were discharged and followed-up for a mean period of 43.8 months (median 28.3, ranging from 0.6 to 144 months). The mortality rate after hospital discharge was 29.3% (29 patients), and deaths occurred within 3.94 and 164.5 months.

### Survival Curves

We constructed Kaplan-Meier survival curves considering the occurrence of total deaths (due to cardiac and non-cardiac causes) and applied a log-rank (Mantel-Cox) test for comparing them.

#### Total survival

Fifty-three (43.0%) patients died during the 43.8-month follow-up; 24 patients died in the hospital and 29, after discharge. The annual mortality rate was 11.8% and 0.52 per 1000 procedures/year. Figure 2 represents the total survival curve for this study.

**Figure 2:** – Survival curve for the studied cases. X-axis: time (months); Y-axis: cumulative survival probability.

#### Sepsis

Out of the 23 patients diagnosed with sepsis, 15 (65.2%) died during the 43.8-month follow-up (p < 0.0001 in the log-rank test, Figure 3). Analyses with 6- and 36-month follow-up periods yielded the same p-value.

**Figure 3:** – Survival curve regarding sepsis. X-axis: time (months); Y-axis: cumulative survival probability. Blue curve: patients who did not have sepsis during hospitalization; green curve: patients who progressed to sepsis during hospitalization.

#### Other variables

No differences were observed regarding sex (p = 0.89) and etiologic agent (p = 11). As for device types, the mortality rate was 48.8% in patients with PM, 29.2% in patients with ICD, and 27.2% among those with CRT devices (p = 0.92). Among patients who presented endocarditis during their hospitalization, 47.8% died during the 43.8-month follow-up (p = 0.93), with no significant differences even when considering 6- and 36-month follow-up periods (p = 0.11 and 0.08, respectively). Considering ejection fractions < 50% or ≥ 50%, the death rate was 44.2% and 41.5% during the whole follow-up (p = 0.06). As for treatment modalities, 42.8% of patients treated only with antibiotics died, while 18.2% of those subjected to partial removal of the system and 47.7% of those subjected to complete removal died (p = 0.07). Considering implant types, 32.8% of patients who underwent endocardial reimplantation and 52.2% of those who underwent epicardial reimplantation died (p = 0.04).

## Comparison between Patients with and without Chagas’ Disease

When comparing these 2 groups, no differences were observed regarding variables (age, sex, device type, number of procedures, time between the last implantation and the diagnosis of infection, leukocytes, C-reactive protein, left ventricular ejection fraction, length of stay, infectious agent, extrusion of the generator, proportion of infectious endocarditis and sepsis, and treatment modality). No differences were observed in mortality either (intra-hospital and after discharge).

## Discussion

CIED implantation increased significantly in recent years owing to broader indications for these devices, to an increasing life expectancy, and to a higher number of people with heart disease. CIED-related infection represents a severe problem, with high morbidity and mortality indices and a great socioeconomic impact due to the high cost of its treatment.^7,18,19^

In this study, the mean patient age was similar to that observed in other studies,^9,13^ as was the predominance of male sex among patients with CIED-related infection.^9,20,21^The main etiology of baseline heart diseases in this study was Chagas’s disease, which differed from that observed in other countries where ischemic heart disease is more prevalent.^4,9^

Infection rates may vary according to the follow-up period, device type, and procedure.^5,21^ This study revealed that a higher proportion of infections was related to generator replacement, device upgrade, and pocket revision. The mean time between the last manipulation and diagnosis of infection, according to the literature, is 20 months,^13^ which is similar to this study but may vary depending on the device. This interval was 4.2 months in relation to the infection of the ICD, according to the literature.^22^

As for infection etiology, staphylococci cause most of CIED-related infections, being responsible for 60% to 80% of the reported cases;^1,23^ this rate is higher than that observed in this study. However, a high number of blood cultures provided negative results, which is unlike what is commonly reported in the literature.^1,5,24^ This difference may be attributed to previous use of antibiotics by the patients (before hospitalization).^25^

For diagnosing this infection, apart from the clinical method, laboratory and echocardiogram examinations are indicated. The transesophageal echocardiogram is the most indicated examination for diagnosing endovascular infection owing to its 88% sensitivity and 99% specificity. Transthoracic echocardiogram, in turn, presents a sensitivity of only 32%.^3^ Therefore, despite the high sensitivity of this imaging test and its precise indication in this picture, clinical correlation and blood culture results are fundamental for the diagnosis and complications of this type of infection. One of these complications is endocarditis, a severe infection that may occur in 0.06% to 7.0% of CIED-related infections,^13^ with an annual incidence of 1.83 cases/million people and 390 cases/million PM recipients,^26^ and a reported mortality of up to 26%.^27^ In the studied population, 57.7% of the patients developed endocarditis. In agreement with the literature, this study demonstrated worsening prognosis in those who had endocarditis. Another complication was sepsis, which also contributed to a high number of deaths; according to the literature, the death rate due to sepsis can vary from 32.2% to 51.1% and its main agent is *S. aureus*.^28,29^The tendency for an inverse association between endocarditis and extrusion of the generator in this study may derive from the number of patients with endocarditis and extrusion, resulting in a confounding bias since extrusion may or may not be present in cases of endocarditis.

Studies recommend the use of vancomycin as a priority in the beginning of empiric antibiotic therapy when treating CIED-related infections until blood culture results are obtained.^1^In agreement with the literature, in this study vancomycin was used in 73.9% of the cases. In addition to antibiotic therapy, other additional treatment modalities are available, such as the early and complete removal of the system, which has a favorable impact on patient progression and is associated with better survival.^11^ In this study, we observed the benefits of complete system removal aiming to cure the infection without relapse. However, complete removal of the system sometimes involves more complex surgery such as cardiotomy, which may worsen the patient’s clinical picture. Data in the literature demonstrate that a quick device and electrode removal, associated with proper antibiotic therapy and reimplantation of a new epicardial or contralateral device, resulted in a high cure rate with a low risk of operative mortality and recurrent infection.^30^ The percutaneous technique of electrode extraction presents less risk. However, mortality can reach 1.2% in experienced centers due to bleeding, vascular perforations, and cardiac tamponade.^31^

CIED-related infection can result in prolonged hospitalization, which is extended in 13% in comparison to the hospitalization for device implantation.^32^Treatment with antibiotics, extraction and reimplantation, and associated complications contribute to this increase in hospitalization time, which also brings an economic impact. Mean length of stay in this study was 35.5 days. Literature reports indicate a mean stay of 17 days.^22^ This difference can be explained by a higher proportion of patients with infectious endocarditis, which resulted in longer antibiotic treatment as recommended by the literature.^3,15^

In addition to morbidity, CIED-related infection also presents mortality, both intra-hospital and after discharge. Intra-hospital mortality varies widely, according to the literature, depending on the number of patients, older age, and the presence of comorbidities and complications during treatment; it can range from 6% to 14%, while total mortality is approximately 20% in one year,^1,6,9^ reaching 26.9% during a 5-year follow-up.^1,8,33^ In the studied population, the intra-hospital mortality rate was higher than the rates reported in the mentioned studies, which could be justified by a higher number of patients who developed endocarditis and sepsis. Regarding the post-discharge period, studies with follow-up periods of up to 2 years showed that the total death rate can be substantial, varying from 6% to 35%.^34,35^In this study, the post-discharge mortality rate was 23.5% during the 43.8-month follow-up, with an annual rate of 14.5%, which was within those values described in the literature.

As previously described, some variables are associated with unfavorable outcomes and mortality predictors.^1,10-13,21^In our study, no significant association (according to the Kaplan-Meier curves) of survival with device type, infectious endocarditis during hospitalization, and treatment modality was observed. However, there was a significant difference in sepsis complication during hospitalization, with lower survival after discharge, as well as among those who underwent epicardial implantation.

Considering treatment modalities, Kim et al.^9^ reported that patients treated conservatively, ie, only with antibiotic therapy, presented high death rates within a mean time of 25 days.^9^In addition, some studies indicate that early device removal was associated to higher patient survival.^2,36^ When total device removal does not happen, mortality can increase up to 7-fold within 30 days.^3^ A recent study considering 6859 patients with no CIED-related infection compared the progress of patients subjected to extraction and those with abandoned electrodes, demonstrating that electrode removal was associated to a lower infection rate in a 5-year period, but no impact on patient survival was observed.^37^ In a related manner, but with a population that included patients with CIED-related infection, a case-control study demonstrated similar mortality rates for patients with and without infection.^34^ This reflects the heterogeneity of study cases when it comes to clinical profile, time of diagnosis and intervention, and comorbidities; these variables interfere with survival, among other factors. Moreover, a study published in June 2019 with the participation of 62 countries demonstrated that only 39.9% of professionals executing CIED implantation performed pocket irrigation with antibiotics and 44% administered prophylactic antibiotics, with complete removal of the system in 62% of the times in case of infection,^38^ which illustrates the disparity in approaches to patients with CIED-related infection.

As for the epicardial implant, a study comparing PM electrode reimplantation after infection demonstrated a 3.6-fold risk of late andocarditis or device reintervention in 65 patients undergoing epicardial access when compared to 37 patients undergoing temporary PM and subsequent endocardial reimplantation.^39^ This was explained by complications associated with epicardial reimplantation.

The etiology of heart disease in patients with CIED influences its progression. The prognosis of patients with chronic Chagas’ heart disease is unfavorable when compared to other etiologies.^40^ No specific studies on CIED-related infection and chagasic etiology are present in the literature, except for a study on microbial diagnosis with fluid culture.^41^ When comparing 15 patients with infection and 68 without CIED-related infection, with a total of 19 patients with Chagas’ disease, no difference was observed between groups regarding this etiology. In this study, with 55 patients with Chagas’ disease, no differences were observed between variables and regarding progression when comparing patients with and without this disease.

### Study Limitations

The retrospective nature of the study was a disadvantage considering a lower availability of adequate medical records, in addition to a sub-notification of patients with CIED-related infection. This may have affected the infection rate, bringing some bias to the analysis. Moreover, due to the long period of patient inclusion, echocardiography techniques and equipment have changed over time, with no uniformity in this examination and preventing the verification of the affected valve in case of infected endocarditis. Since not all patients underwent transesophageal echocardiography, the rate of endocarditis may have been underestimated.

## Conclusions

The rate of infection was 1.9% (1.2 per 1000 procedures/year), with a predominance of men and patients with dilated cardiomyopathy. During hospitalization, the incidence of infectious endocarditis was 57.7% and that of sepsis was 18.7%. Total system removal was performed in most patients (85.4%). Intra-hospital mortality rate was 19.5% and was associated with the occurrence of endocarditis and sepsis. After discharge, the annual mortality rate was 11.8%, influenced only by the occurrence of sepsis during hospitalization and by epicardial implantation.
